# Evaluation of GSK5862611, an anti‐Sortilin antibody, in hiPSC‐derived complex cellular models harbouring TDP43 G298S risk variant

**DOI:** 10.1002/alz70855_096500

**Published:** 2025-12-23

**Authors:** Thomas Westergard

**Affiliations:** ^1^ GSK, Collegeville, PA, USA

## Abstract

**Background:**

Reduced levels of progranulin (PGRN) protein are associated with frontotemporal dementia (FTD), Alzheimer's disease (AD), Parkinson's disease (PD), and amyotrophic lateral sclerosis (ALS). Sortilin (SORT1) is a scavenger receptor responsible for the uptake of PGRN into the cell targeting its degradation. GSK5862611 (S60‐11) is an anti‐Sortilin (SORT1) human IgG1 antibody developed to inhibit binding of PGRN to sortilin receptors and deplete sortilin receptors from the cell surface, resulting in an increase in extracellular PGRN levels. Similar anti‐sortilin antibodies are currently in phase 2 and phase 3 clinical trials for AD and FTD‐*GRN*, respectively. Given that PGRN plays an important role in maintaining lysosomal functions and neuronal survivability we evaluated whether enhancing PGRN levels could be effective in reversing phenotypes associated with TDP43 G298S risk variant in complex hiPSC‐derived cellular models.

**Method:**

hiPSC‐derived bi‐ and tricultures comprising spinal motor neurons and astrocytes carrying TDP43 risk variant G298S mutation and wild‐type or *GRN* edited microglia were established and treated with GSK5862611, isotype control hIgG1, or recombinant PGRN. Impacts on neurite length and TDP43 mislocalization in motor neurons as well as levels of PGRN, neurofilament light chain (NfL), and glial fibrillary acidic protein (GFAP) in the culture media were assessed.

**Results:**

Recombinant PGRN rescued the loss of neuritic length and TDP‐43 mislocalization in hiPSC‐derived bicultures from TDP43 G298S risk variant. Like PGRN, GSK5861611 also rescued loss of neuritic length and TDP43 mislocalization compared to untreated bicultures, however the effect was not significant when compared to isotype control hIgG1. In hiPSC‐derived tricultures including *GRN* edited microglia, GSK5862611 increased extracellular PGRN levels and reduced TDP43 mislocalization in a dose‐dependent manner. Additionally, GSK5862611 reduced NfL and GFAP levels in TDP43 tricultures with PGRN Het microglia (HumIgG1 had similar reduction in NfL).

**Conclusion:**

Taken together, these results indicate that blocking Sortilin receptors increases extracellular PGRN levels while reducing TDP43 mislocalization in motor neurons, and reducing NfL and GFAP levels in complex hiPSC cellular models carrying a TDP43 G298S risk variant. These data support the hypothesis that increasing PGRN levels with an anti‐sortilin antibody may be a promising therapeutic strategy in ALS.

Acknowledgements: S60‐11 anti‐sortilin mAb was provided by Alector

